# *Helicobacter pylori* induced Immune Thrombocytopenic Purpura and perspective role of *Helicobacter pylori* eradication therapy for treating Immune Thrombocytopenic Purpura

**DOI:** 10.3934/microbiol.2021018

**Published:** 2021-09-02

**Authors:** Arham Ihtesham, Shahzaib Maqbool, Muhammad Nadeem, Muhammad Bilawal Abbas Janjua, Omaima Sundus, Ali Bakht Naqqash, Waleed Inayat Mohamed, Syed Turab Haider, Muhmmad Ahmad, Mir Ahmad Talha Mustafa, Hafiz Osama Mehboob

**Affiliations:** 1 House officers Rawalpindi Medical University, Pakistan; 2 Consultant Pediatrician, THQ Kabirwala, Pakistan; 3 House officers Nishtar Medical University, Pakistan; 4 House officers Services Institute of Medical Sciences, Pakistan; 5 Medical Officer Holy Family Hospital Rawalpindi, Pakistan

**Keywords:** *Helicobacter pylori*, *H-pylori* eradication therapy, Immune thrombocytopenic purpura

## Abstract

Immune thrombocytopenic purpura (ITP) is an autoimmune disease characterised by production of autoantibodies against platelet surface antigens. Recent studies have demonstrated a paramount association of ITP and *Helicobacter pylori (H-pylori)* infection with significant rise in platelet count following *H-pylori* eradication therapy. The *H-pylori* infection induced ITP is validated by many proposed mechanisms such as molecular mimicry due to production of autoantibodies against *H-pylori* surface virulent factors (CagA) and cross reactivity of these antibodies with platelet surface antigens (GP IIb/IIIa, GP Ib/IX, and GP Ia/IIa), phagocytic perturbation due to enhanced phagocytic activity of monocytes, enhanced dendritic cell numbers and response, platelets aggregation due to presence of anti- *H-pylori* IgG and von Willebrand factor (vWf) and finally host immune response against *H-pylori* virulent factors CagA and VacA leading to ITP. The effectiveness of *H-pylori* eradication therapy has also been demonstrated with platelet count being used as a predictive factor for assessment of treatment efficacy. Out of 201 patients 118 were responding to the triple therapy and remaining 83 patients were non-responders, showing the response rate of 58.7%. Out of 118 responders 69 patients were showing complete response (CR) and 49 were showing partial response (PR) to the *H-pylori* eradication therapy. However, more studies are required to elucidate this association and treatment efficacy.

## 1. Introduction

Immune thrombocytopenic purpura (ITP) is an autoimmune disease characterized by the production of autoantibodies against platelets membrane antigens leading to platelets destruction by the Reticuloendothelial system [1],[2]. The normal adult platelets range from 150–400 × 10^9^/L of blood with a normal life span of 8–10 days. The formation of autoantibodies and immunocomplexes in the blood leads to a reduction in platelet count to 100 × 10^9^/L or less [3]. The pathophysiology associated with the development of immune thrombocytopenic purpura is the formation of IgG antibodies against platelets surface proteins like GPIIb-IIIa and GIb-IX; however, many unknown mechanisms need to be explored in the pathogenesis of ITP [4].

ITP is classified as acute, persistent and chronic. The acute ITP lasts for 3-months, most commonly presents in children but resolves spontaneously without any therapy. The persistent type of ITP lasts for 3–12 months and the chronic form lasts for more than 12-months. The chronic form of ITP is commonly seen in adults that may persist but can resolve in 20–40% of the patients later. [5]. The etiological classification of ITP ranges from primary ITP with no identifiable cause to secondary ITP having secondary association with environmental factors, neoplastic disorders, bacterial and viral infections like hepatitis C virus (HCV), and human immunodeficiency virus (HIV), and chronic *H-pylori* infection [6].

*Helicobacter pylori* (*H-pylori*) is a gram-negative, spiral-shaped, flagellated, microaerophilic bacillus that resides inside the stomach and is transmitted through fecal-oral, oral-oral route [7]–[9]. The prevalence of *H-pylori* is high in developing countries and is known to affect more than 50% of the world population either clinically or asymptomatically [9]. *H-pylori* are recognized as a causative agent in the development of gastritis, peptic ulcer disease, gastric atrophy and poses an increased risk of gastric adenocarcinoma and mucosal-associated lymphoid tissue lymphoma (MALT) [10]. *H-pylori* infection is also known to be associated with non-gastrointestinal diseases like coronary artery disease, pernicious anemia, ITP, and various other autoimmune disorders as well [11]–[13]. ITP is a diagnosis of exclusion, where the underlying cause of its development is unknown according to various literature. The pathophysiological link between *H-pylori* and ITP was initially demonstrated by Gasbarrini et al., Who demonstrated the effectiveness of *H-pylori* eradication therapy in improving platelet count in patients with chronic ITP [13].

The objective of our study is to demonstrate how *H-pylori* is associated with immune ITP and how effective *H-pylori* eradication therapy is in improving platelet count in patients with ITP.

## 2. Materials and methods

### 2.1. Search strategy

The study was conducted according to preferred reporting items for systematic reviews and Meta-analyses (PRISMA) guidelines [14]. The authors did a well-organized data search through various databases like PubMed, Medline, EMBASE, Web of Sciences, and Google scholar. The search terms were *‘Helicobacter pylori’* ‘immune Thrombocytopenic purpura’ ‘*Helicobacter pylori* eradication therapy,’ and ‘treatment of immune Thrombocytopenic purpura.’

### 2.2. Inclusion and exclusion criteria

(1) All those studies involving diagnosed cases of chronic ITP, according to the American Society of Haematology (ASH) as given below [15], are included in our study.

- Platelets count less than 100 × 10^9^/L.
- Exclusion of secondary causes of ITP (Drugs, HCV, HIV).
- Examination of blood smear of all patients.

(2) All those studies carried out on *H-pylori* infection diagnosed by reliable tests like urea breath test (UBT), serological test for *H-pylori*, stool antigen tests, and gastric mucosal biopsy for histological diagnosis were included in our study.

(3) All those studies involving those cases of ITP who are also infected with *H-pylori* infection and delineating the response of *H-pylori* eradication therapy in patients with ITP in terms of improved platelets count were included.

(4) All study types like observational, clinical trials, review articles, meta-analysis were included, but all those reports published only in abstract form were excluded from our study criteria.

(5) All those studies on diagnosed cases of ITP and were receiving platelet transfusions were also excluded from our study criteria. Similarly, those studies involving the use of *H-pylori* eradicating programs before screening for *H-pylori* infection were excluded as well.

### 2.3. Data extraction

Data extraction from eligible studies was undertaken by two independent reviewers (AI and SM) and after carefully screening the eligible studies, the data deemed suitable to support our study objectives was included. During the screening of eligible studies, a third investigator (MNL) was also consulted to make the data more reliable with clear-cut shreds of evidence to support our objectives. The data in terms of first author names, year of publication, diagnostic criteria of ITP and *H-pylori*, treatment of ITP through *H-pylori* eradication, and treatment response in improving platelets count was extracted from eligible studies.

### 2.4. Quality assessment

The quality of each study included in our review was assessed by using Jadad five-item scale for RCTs. A final score was 0–5, with ≤2 representing poor quality and ≥3 represent good-quality studies. And the quality of case-control and cohort studies was assessed through the Newcastle-Ottawa scale (NOS).

## 3. Search result

**Figure 1.**
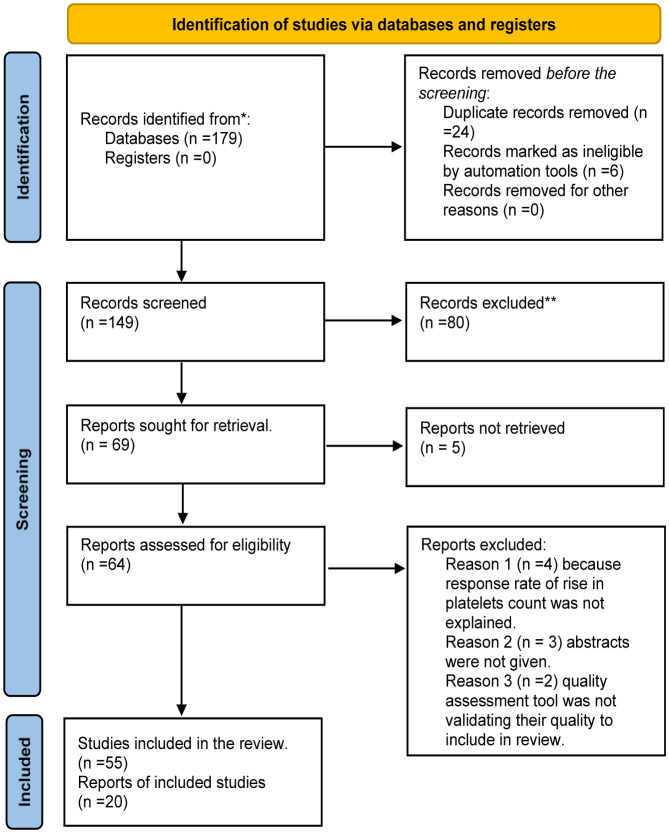
PRISMA Flow chart for selection of studies. * Reporting the number of records identified from each database or register searched (rather than the total number across all databases/registers). ** Indicate how many records were excluded by a human and how many were excluded by automation tools.

After searching through various search engines, 179 articles were retrieved, and after careful screening of abstracts and titles, 110 articles were excluded. Finally, 55 articles were included in our study after excluding 14 articles that did not provide us information related to our inclusion criteria. The schematic diagram of selected studies is given below in Figure 1.

## 4. Pathophysiology of Helicobacter-Pylori infection-induced Immune Thrombocytopenic Purpura

**Figure 2.**
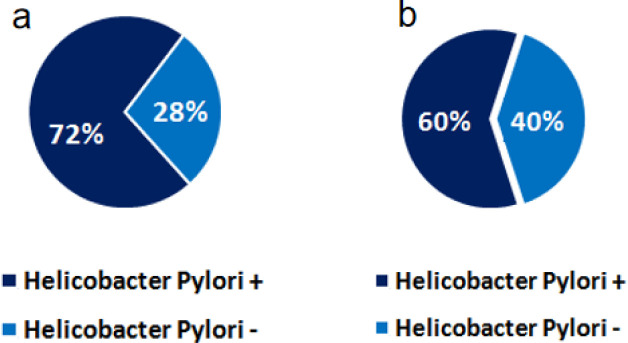
a: The prevalence of *H-pylori* antigen positivity in patients with chronic ITP. The data is extracted from a study by Aljarad et al. conducted on 50 patients with Chronic ITP [16]. b: The prevalence of *H-pylori* surface antigen positivity in patients with chronic ITP. This data is extracted from Sheema et al. conducted on 85 diagnosed patients of ITP through reliable diagnostic tools [17].

The ostensible association between *H-pylori* and ITP was initially described in 1998 by Gasbarrini et al. when he observed the rise in platelet count in patients of ITP when they were treated with *H-pylori* eradication therapy [13]. This evidence of rise in platelet count after *H-pylori* eradication therapy leads to the concept of involvement of *H-pylori* as a secondary cause of immune thrombocytopenic purpura. A similar study by Aljarad et al. involving 50 diagnosed patients of chronic ITP also demonstrated the association of *H-pylori* infection in about two-third (n = 36) of the patient's as shown in Figure 2a who were treated for ITP, which further validates the ostensible link between *H-pylori* infection and ITP [16]. In the same vein, another study by Sheema et al. has also demonstrated the prevalence of *H-pylori* surface antigen positivity in patients of chronic ITP, as shown in Figure 2b [17]. There are many proposed mechanisms of ITP development due to *H-pylori* infection, such as molecular mimicry, platelets aggregation, phagocytic perturbation, increased plasmacytoid dendritic cell (pDCs) response and host immune response to *H-pylori* virulent factors [6].

### 4.1. Molecular mimicry

The production of antibodies against *H-pylori* antigens such as cytotoxin-associated gene A (CagA) causes cross-reactivity against various glycoproteins antigens (GP IIb/IIIa, GP Ib/IX, and GP Ia/IIa) present on platelets membrane [18]. This mechanism of cross-reactivity was also observed in patients with Acquired Immune deficiency Syndrome (AIDS) caused by HIV that is also being recognized as a secondary cause of ITP. The production of antibodies against various glycoproteins like HIV gp24 and gp120 in HIV-infected patients are also known to react with platelets membrane antigens to the presence of similar epitopes [19],[20].

### 4.2. Phagocytic perturbation

Another proposed mechanism of *H-pylori* infection-induced ITP is through inhibition of the Fcγ receptors on peripheral blood monocytes caused by *H-pylori* infection, leading to increased anti-platelets antibodies with increased platelets turnover due to reduced production of FcγRIIB [21]. The reduced expression of FcγRIIB and the emergence of autoreactive B-cells cause increased phagocytic activity of monocytes and reduction in platelet count [21],[22].

### 4.3. Dendritic cells response to HP infection

Another proposed mechanism of *H-pylori* induced ITP is increased numbers of plasmacytoid dendritic cells having the exquisite role of antigen-presenting cells [23]. The extension of lamina podia of dendritic cells into the intact gastric epithelium through the paracellular pathway causes exposure to *H-pylori* antigen and ultimately enhanced host immune response against *H-pylori* antigens through Th1 and Th2 lymphocytes leading to the production of IL-12 and IL-10. The presence of outer membrane proteins (Omp's) such as recombinant HpaA (rHpaA) and recombinant outer membrane protein 18 (rOmp-18) on the *H-pylori* surface stimulates the production of IL-12 and IL-10 from dendritic cells because of their antigenic potential [23],[24].

### 4.4. Platelets aggregation

Another proposed mechanism of *H-pylori* induced ITP is through platelet aggregation caused by some strains of *H-pylori*. The presence of anti- *H-pylori* IgG and von Willebrand factor (vWf) on cell membranes of various *H-pylori* strains causes platelet activation and aggregation [18]. The presence of von Willebrand factor on cell membranes of *H-pylori* causes platelets to aggregate through glycoprotein-Ib (gp-Ib) present on platelets surface; similarly, anti- *H-pylori* IgG interaction with IgG receptors (FcgRIIA) on platelets surface also causes platelets to aggregate. The binding of vWf with gp-Ib causes activation of signalling pathway intracellularly, leading to activation of gp-IIb/IIIa and irreversible binding to platelets to vWf [25]. Similarly, *H-pylori* induced platelet aggregation in gastric microvasculature with the manifestation of systemic-onset disease is another proposed mechanism of *H-pylori* induced ITP [18].

### 4.5. Host immune response and Helicobacter-pylori infection

Various studies have reported the association of *H-pylori* with various outer membranes proteins (Opm's) like outer inflammatory protein A (OipA), blood group antigen-binding adhesion A (BabA), and sialic acid-binding adhesion (SabA) with the exquisite role of *H-pylori* binding with gastric epithelium [26],[27]. In the same vein, studies have also reported the association of *H-pylori* with various virulent factors such as cytotoxin-associated gene A (CagA) and vacuolating associated gene A (VacA), which help in colonization and infection [28].

The CagA is known to be located in a 40Kb cluster of terminal genes on cytotoxin antigen pathogenicity island (Cag PAI) that codes for the production of Cag A proteins and type IV secretion system (T4SS) [29]. Those infected with the Cag PAI^+^ strain of *H-pylori* are more likely to develop gastric ulceration and gastric carcinoma than those with Cag PAI-negative strain. The type IV secretory system (T4SS) acts as a transport vehicle for transporting CagA proteins from gastric mucosal surface to endothelial cells where CagA protein is tyrosine-phosphorylated at the site containing Glu-Pro-Ile-Tyr-Ala (EPIYA) sequence and initiate strong host immune response by induction of IL-8 (a pro-inflammatory cytokine) and NF-KB mediated immunoinflamatory response [30],[31]. Soon after immune system activation, the host immune system starts producing anti-CagA antibodies (IgG) with a strong affinity for platelets surface glycoproteins (GP IIb/IIIa, GP Ib/IX, and GP Ia/IIa) through the mechanism of cross-reactivity and platelets destruction and clearance by Reticuloendothelial system (RES) [18].

Similarly, the role of VacA (the second most important virulent factor of *H-pylori*) is also of paramount importance in the pathogenesis of *H-pylori* induced ITP. The studies have shown the exquisite role of VacA in blocking T-Helper cells by an interruption in the T-cell receptor IL-2 pathway [32]. In the same vein, the binding of VacA with multimerin-1, a massive, soluble, disulfide-linked homopolymeric protein also called elastin microfibril interfacer 4 (EMILIN-4) expressed on megakaryocytes and platelets encoded by the MMRN1 gene, enhances the platelets activation and clearance [33],[34]. The role of genetic factors such as HLA-class II allele patterns has also been demonstrated in the pathogenesis of *H-pylori* induced ITP but still, very rare work has been done so far to accept this fact as a generalized mechanism [35].

## 5. Helicobacter-Pylori Eradication therapy and Immune thrombocytopenic Purpura

Although the exact mechanism of *H-pylori* induced ITP is not conclusively elaborated, it is now considered standard practice to test for *H-pylori* infection in the face of ITP. In the year 2010, many experts from different countries concluded in a consensus report that ITP is one of the extragastric manifestations of Helicobacter pylori infection and a strong indication for *H-pylori* eradication therapy [36]. According to guidelines of the American society of Hematology (2011), *H-pylori* infection is acknowledged as a secondary cause of ITP and recommended the testing for *H-pylori* infection in patients with ITP and living in endemic areas [37]. Similarly, in 2009, the Asia-Pacific conference about the management of *H-pylori* infection also concluded the use of *H-pylori* eradication therapy for the treatment of ITP [38].

The major complication associated with ITP individuals is life-threatening hemorrhage, particularly intracranial hemorrhage [39]. The treatment of ITP is recommended when the platelet count becomes less than 50 × 10^9^/L and in patients undergoing surgery or suffered trauma [40]. According to the American society of Hematology, the currently known treatment for patients of ITP is through Intravenous immunoglobulin (IVIG), corticosteroids, immunosuppressive therapy, anti-D immunoglobulin, and splenectomy. Similarly, according to the revised guidelines of ITP management by ASH (2019), the role of rituximab, eltrombopag (Revolade) and romiplostim have also been implicated in treatment of ITP [37]. In the same vein, the *H-pylori* eradication therapy consisting of triple therapy like Proton pump inhibitors (omeprazole, lansoprazole, pantoprazole) and antibiotics like amoxicillin, clarithromycin, and metronidazole for two weeks is also now recommended for patients of ITP as a long-term treatment [34]. The effectiveness of triple cocktails in patients of ITP is shown in (Table 1).

**Table 1.** Showing rise in platelet count in response to *Helicobacter pylori* eradication therapy.

Authors Name	year of study	Country of study	Diagnostic criteria *H-pylori* detection	Pre-Treatment platelets count	Treatment given	Post-treatment platelets count
Maghbool et al. [41]	2009	Iran	All patients with positive stool antigen test (ELISA) for *H-pylori* infection. All patients were diagnosed cases of ITP.	The median platelet count was 18.6 × 10^9^/L.	Triple Therapy (omeprazole 1 mg/kg/day, metronidazole 30 mg/kg/day, amoxicillin 60 mg/kg/day) for 14 days and follow-up for one year.	Median platelets count increased to 79.2 × 10^9^/L after a one-year follow-up.
Goto et al. [42]	2001	Japan	A 53 years old female known case of ITP with positive *H-pylori* infection on GI-endoscopy (showing superficial gastritis) and positive rapid urease test and positive histology for *H-pylori* infection.	The platelet count before *H-pylori* eradication therapy was 24 × 10^9^/L.	Triple therapy (amoxicillin 1000 mg twice daily, clarithromycin 500 mg twice daily, lansoprazole 60 mg once per day) for 14 days.	Following treatment with triple therapy, the platelets count increased from 24 × 10^9^ to 134 × 10^9^/L.
Hwang et al. [43]	2016	Korea	All 102 diagnosed patients of chronic ITP had 39 patients positive for *H-pylori* infection on the C-13 urea breath test (UBT).	The platelets count before eradication therapy was 40.3 ± 29.1 ×10^3^/µL (40.3 ± 29.1 × 10^9^/L) for patients with HPPE group (n = 39).	Triple therapy (rabeprazole 20 mg twice daily, amoxicillin 1000 mg twice daily, clarithromycin 500 mg twice daily) for 7 days.	The platelets count after 2-months of follow-up following eradication therapy was 104.1 ± 47.4 × 10^3^/µL, and that rose to 155.3 ± 68.7 × 10^3^/µL (155.3 ± 68.7 × 10^9^/L) after 6-months of follow-up in patients with the HPPE group.
Veneri et al. [44]	2002	Italy	All patients with diagnosed cases of ITP and positive for *H-pylori* infection on biopsy and UBT.	The platelets count before eradication therapy was 51.6 × 10^9^/L.	Triple therapy (amoxicillin 1000 mg twice daily, clarithromycin 500 mg twice daily, pantoprazole 40 mg once per day) for 7-days.	Following treatment with *H-pylori* eradication therapy, the platelets count increased to 143.3 × 10^9^/L with a P-value of 0.01.
Aljarad et al. [16]	2018	Syria	All 50 patients diagnosed cases of chronic ITP were diagnosed with *H-pylori* infection. Out of 50 patients, 36 were diagnosed with *H-pylori* infection based on biopsies taken by EGD and Urea breath test (UBT).	At the beginning of the therapy, the mean value of platelets was 46.25 ± 17.7 × 10^9^/L in patients with *H-pylori* positive group (n = 36). Similarly, the mean value of platelet count in patients with the *H-pylori* negative group was 25.21 ± 8.4 × 10^9^/L.	Triple therapy (omeprazole 40 mg once per day, amoxicillin 1000 mg twice daily, clarithromycin 500 mg twice daily) for 14 days.	Following treatment at the end of the first month, the mean value of platelet count was 67.9 × 10^9^/L in patients with *H-pylori* positive group compared to 28.28 × 10^9^/L in *H-pylori* negative patients. At the end of the third month, the mean platelets count was 112.13 × 10^9^/L in *H-pylori* ^+^ patients, and at the end of the sixth month, the mean platelets count in *H-pylori* ^+^ patients were 98.66 × 10^9^/L.
Samson et al. [45] (case-1)	2012	Netherland	A 75 years old male known case of ITP and positive for *H-pylori* infection diagnosed through UBT.	Before the start of the eradication therapy, the platelet count was 7000 cells/mL of blood.	The patient was given corticosteroids and immunoglobulins, but no improvement was observed. The patient was also given triple therapy (omeprazole 40 mg once a day, amoxicillin 1000 mg twice daily, clarithromycin 500 mg twice daily).	After four months of follow-up following *H-pylori* eradication therapy, the platelets count increased from 7000 cells/mL to 140000 cells/mL, and UBT also becomes negative.
Samson et al. [45] (case-2)	2012	Netherland	A 47 years old male presented with renal colic, and an incidental diagnosis of ITP was made. The test for detection of *H-pylori* infection (UBT) was positive.	At the start of treatment patient's platelet count was 15000 cells/mL of blood.	The patient was given triple therapy (omeprazole 40 mg once a day, amoxicillin 1000 mg twice daily, clarithromycin 500 mg twice daily). The patient was also given quadruple therapy after the relapse of the disease.	Following triple therapy, platelets count increased up to 100000 cells/mL within one and a half month with relapse after few months with platelets count falls to 51000 cells/mL, and UBT was also positive. However, following quadruple therapy, platelets count become 125000 cells/mL of blood within 5 months and remain stable after UBT being negative.

**Note: ***H-pylori*: *Helicobacter pylori*, UBT: Urea breath test, ITP: Immune thrombocytopenic purpura, EGD: Esophagogastroduodenoscopy, ELISA: Enzyme-linked immunosorbent assay. HPPE group: *H-pylori* positive eradication group.

**Table 2.** Showing platelet response towards *Helicobacter pylori* eradication therapy.

Authors Name	Year of Study	Country of Study	Prevalence of *H-pylori* Infection	Detection of *H-pylori* Infection	Treatment Given	Platelets Response
Maghbool et al. [41]	2009	Iran	Out of thirty diagnosed cases of ITP, five were found positive for *H-pylori* infection, with a prevalence of 17%.	All patients with positive stool antigen test (ELISA) for *H-pylori* infection.	Triple Therapy (omeprazole 1 mg/kg/day, metronidazole 30 mg/kg/day, amoxicillin 60 mg/kg/day) for 14 days and follow-up for one year.	All the patients diagnosed with *H-pylori* infection (n = 5) showed improvement in platelet count after triple therapy with a 100% response rate (5/5).
Jaing et al. [46]	2003	Taiwan	Out of twenty-two diagnosed cases of ITP, nine were found positive for *H-pylori* infection, with a prevalence of 41%.	All patients with positive stool antigen test (ELISA) for *H-pylori* infection.	Triple therapy (amoxicillin 1000 mg twice daily, clarithromycin 500 mg twice daily, lansoprazole 60 mg once per day) for 7 days.	Of all the patients diagnosed with *H-pylori* infection (n = 9) who received eradication therapy, only 5 patients showed a rise in platelet count with a response rate of 55.5%, and platelets remain elevated throughout the follow-up period.
Ando et al. [47]	2003	Japan	Out of 61 diagnosed cases of ITP, 50 patients were found positive, with a prevalence of 83%.	*H-pylori* detection was done through a urea breath test (UBT).	Triple therapy (amoxicillin 1000 mg twice daily, clarithromycin 500mg twice daily, pantoprazole 40 mg once per day) for 7-days.	Out of 50 *H-pylori* positive patients, only 29 patients received *H-pylori* eradication therapy, and 27 patients with a response rate of 93% showed a rise in platelet count with UBT being negative. Similarly, 16 (55.1%) out of 29 patients showed a major or minor response.
Hashino et al. [48]	2003	Japan	Out of 22 diagnosed cases of ITP, 14 were found positive for *H-pylori* infection with a prevalence rate of 64%.	Histological and cultural examination of a biopsy obtained from gastric mucosa through GI endoscopy.	The patient was given triple therapy (omeprazole 40mg once a day, amoxicillin 1000 mg twice daily, clarithromycin 500 mg twice daily) for 7 days.	Out of 14 *H-pylori* positive patients, only 13 patients received *H-pylori* eradication therapy, and only 5 patients showed recovery in platelet count (more than 100 × 10^9^/L) with a response rate of 38.4%.
Tag HS et al. [49]	2010	Korea	Out of 25 diagnosed cases of ITP, 23 patients were found positive for *H-pylori* infection with a prevalence rate of 92%.	*H-pylori* detection was done through UBT, serum *H-pylori* antibodies, and rapid urease test (CLO test).	The patient was given triple therapy (omeprazole 40mg once a day, amoxicillin 1000 mg twice daily, clarithromycin 500 mg twice daily) for 14 days.	Out of 23 diagnosed cases of *H-pylori* infection and received *H-pylori* eradication therapy, only 17 patients were showing response in terms of an increase in platelet counts with a response rate of 73.9%.
Payandeh et al. [50].	2012	Iran	Out of 52 diagnosed cases of ITP, only 35 patients were found positive for *H-pylori* infection, with a prevalence of 67.3%.	*H-pylori* detection was done through UBT and serum *H-pylori* antibodies.	Triple therapy (amoxicillin 1000 mg twice daily, clarithromycin 500 mg twice daily, pantoprazole 40 mg once per day) for 14-days.	Out of 35 diagnosed cases of *H-pylori* infection, only 26 patients received *H-pylori* eradication therapy, but only 15 patients showed a rise in platelet count (more than 100 × 10^9^/L) with a response rate of just 57.7%.
Lee et al. [51]	2020	Korea	Out of 138 diagnosed patients of 75 were found positive for *H-pylori* infection with a prevalence of 54.3%.	*H-pylori* detection was done through UBT, CLO test, Wright Giemsa stain, and *H-pylori* antibodies.	Triple therapy (PPI twice a day, amoxicillin 1000 mg twice daily, clarithromycin 500 mg twice daily) for 7–21 days. And Quadruple therapy (PPI twice a day, bismuth 120 mg four times a day, metronidazole 500 mg three times a day, tetracycline 500 mg four times a day) for 14 days in patients unresponsive to triple therapy.	The complete response rate in the eradication group at the end of two months was 38.5%, and at the end of the sixth month, it was just 36.8%. However, patients who achieved CR at 2 months of therapy showed a sustained response in 77.8% of the patients.

**Note:** *H-pylori*: *Helicobacter pylori*, UBT: Urea breath test, ITP: Immune thrombocytopenic purpura, EGD: Esophagogastroduodenoscopy, ELISA: Enzyme-linked immunosorbent assay. HPPE group: *H-pylori* positive eradication group.

## 6. Response criteria for rise in platelet count following eradication therapy

Various studies have reported variable response criteria in terms of complete, partial and no response. In a study by Payandeh et al. [52], the response criteria defined was as followed:

- **Complete response (CR):** when platelet count become >150 × 10^3^/µL of baseline count at 6 months of treatment.
- **Partial response (PR):** when platelet count rises to >30 × 10^3^/µL of baseline count at 6 months of treatment.
- **No response (NR):** when rise in platelet count follows none of the above given values.

Similarly, according to international working group guidelines on ITP [53], the response criteria was defined as follows:

- **Complete response (CR):** Platelet count of at least 100 × 10^3^/µL at 2 months of follow-up with or without maintenance therapy.
- **Partial response (PR):** Platelet count of at least 30 × 10^3^/µL at 2 months of follow-up or doubling of platelet count over a period of more than 2 months.
- **No response (NR):** Platelet count of less than 30 × 10^3^/µL at 2 months of follow-up or didn't increase above 50% of pre-treatment level at 2 months of treatment.

Most of the studies conducted for assessment of platelet responses after Helicobacter pylori eradication therapies follow this international working group guidelines criteria to categorise platelet response in terms of complete, partial and no response. However, no matter which criteria is being followed, the ultimate goal is the assessment response of platelet count after *H-pylori* eradication therapy to validate the effectiveness of *H-pylori* eradication therapy in treatment of ITP as shown in Table 3.

**Table 3.** Showing the level of response (complete, partial and no response) following eradication therapy.

Authors Name	Year of Study	Country of Study	Study characteristics and cases identified.	Treatment given	Complete Response (CR)	Partial Response (PR)	No Response (NR)
Song et al. [54]	2008	Korea	A total of 34 patients with ITP and *H-pylori* infection identified based on CLO, UBT, and IgG and were given EA and EL were involved in the study.	Triple therapy (Amoxicillin 1 g twice daily, clarithromycin 500 mg twice daily, esomeprazole 40 mg twice daily) for 14 days.	CR was observed in just 4 patients.	PR was observed in 10 patients.	The remaining 20 patients were not showing any response.
Aljarad et al. [16].	2018	Syria	Out of 50 diagnosed cases of ITP, 36 were diagnosed with *H-pylori* infection based on histological examination through biopsies, UBT, and anti- *H-pylori* antibodies.	Triple therapy (omeprazole 40 mg once per day, amoxicillin 1000 mg twice daily, clarithromycin 500 mg twice daily) for 14 days.	CR was observed in 10 patients.	PR was observed in 18 patients.	The remaining 22 patients either given eradicated therapy or not were showing no response platelets count.
Sheema et al. [17]	2017	Pakistan	Out of 85 cases of ITP only 34 patients were found positive for *H-pylori* infection on the basis of stool antigen test through the Rapid Immunochromatography.	Triple therapy (Amoxicillin 1 g twice daily, clarithromycin 500 mg twice daily, proton pump inhibitor 40 mg twice daily) for 14 days.	CR was observed in 19 patients.	PR response was observed in 10 patients.	The remaining 5 patients who were given eradication therapy didn't showed any response
Tag HS et al. [49].	2010	Korea	Out of 25 diagnosed cases of ITP 23 were diagnosed positive for *H-pylori* infection based on UBT, rapid urease test (CLO test) by endoscopic biopsy.	Triple therapy (Amoxicillin 1 g twice daily, clarithromycin 500 mg twice daily, proton pump inhibitor 40 mg twice daily) for 7 or 14 days.	CR was observed in **11** patients.	PR was observed in **6** patients.	The remaining **8** patients who were given eradication therapy didn't showed any response.
Payandeh et al. [50].	2012	Iran	Out of 52 diagnosed cases of ITP, only 35 patients were found positive for *H-pylori* infection based on UBT and antibodies tests, but 3 patients with autoimmune disease, 2 with HBV and 1 with HCV infection were excluded and only 26 were assessed for response.	Triple therapy (Amoxicillin 1 g twice daily, clarithromycin 500 mg twice daily, proton pump inhibitor 40 mg twice daily) for 14 days.	CR was observed in **15** patients.	None of the treated patients showed partial response.	The remaining **11** patients who were given eradication therapy didn't showed any response.
Sato et al. [55]	2004	Japan	Out of 53 diagnosed cases of ITP only 39 were found positive for *H-pylori* infection based on UBT and only 32 patients received eradication therapy.	Triple therapy (Amoxicillin 1 g twice daily, clarithromycin 500 mg twice daily, proton pump inhibitor 40 mg twice daily) for 14 days.	CR was observed in **10** patients.	PR was observed in **5** patients.	The remaining **17** patients who received eradication therapy did not showed any response.

**Figure 3.**
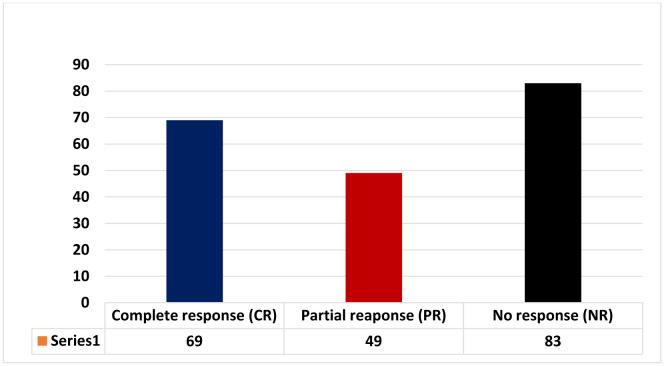
showing complete, partial and no response to eradication therapy.

A total of 201 patients were involved in this review to validate the effectiveness of *H-pylori* eradication therapy and if we categorise these patients into responders (complete or partial response) and non-responders following eradication therapy, we come to know that 118 patients show responsiveness in terms of rise in platelet count following eradication therapy and only 83 patients show no response. The response rate based on complete response, partial response and no response is shown in Figure 3.

## 7. Conclusion

Literature over the past few years has elucidated a paramount association between *H-pylori* infection and development of ITP. Various mechanisms of *H-pylori* induced ITP have been proposed by various researches in literature; however, the most commonly discussed mechanism is the role of molecular mimicry (production of autoantibodies against *H-pylori* virulent factor CagA and cross reactivity of these antibodies with various surface antigens such as GP IIb/IIIa, GP Ib/IX, and GP Ia/IIa, on platelet membranes). The other associated mechanisms are phagocytic perturbation due to increased phagocytic activity of monocytes and down regulation of FcγRIIB receptors, increased production of plasmacytoid dendritic cells which due to their lamina podia activate host immune response and it leads to production of various interleukins. Furthermore, the presence of anti- *H-pylori* IgG and von Willebrand factor (vWf) on cell membranes of various *H-pylori* strains causes platelet activation and aggregation. Finally, the host immune system comes into action with production of antibodies against *H-pylori* virulent factor CagA, and binding of VacA with multimerin-1 on platelet surfaces leading to thrombocytopenic purpura. Similarly, the role of *H-pylori* eradication therapy (triple therapy) has also been demonstrated in patients of ITP with significant rise in platelet count from the baseline of pre-treatment levels. Out of 201 patients from various studies who participated in this review and were treated with eradication therapy, significant response was observed in 118 patients with rise in baseline platelet count validating the effectiveness of triple therapy in treatment of ITP.
